# Endophytic fungal diversity of *Fragaria vesca*, a crop wild relative of strawberry, along environmental gradients within a small geographical area

**DOI:** 10.7717/peerj.2860

**Published:** 2017-01-05

**Authors:** Kazutomo Yokoya, Sarah Postel, Rui Fang, Viswambharan Sarasan

**Affiliations:** Natural Capital and Plant Health, Royal Botanic Gardens Kew, Richmond, Surrey, United Kingdom

**Keywords:** Root, Soil, Stress, Fitness benefit, *In vitro*, Biomass, Beneficial fungus

## Abstract

**Background:**

Fungal endophytes are highly diverse ubiquitous asymptomatic microorganisms, some of which appear to be symbiotic. Depending on abiotic conditions and genotype of the plant, the diversity of endophytes may confer fitness benefits to plant communities.

**Methods:**

We studied a crop wild relative (CWR) of strawberry, along environmental gradients with a view to understand the cultivable root-derived endophytic fungi that can be evaluated for promoting growth and tolerating stress in selected plant groups. The main objectives were to understand whether: (a) suboptimal soil types are drivers for fungal distribution and diversity; (b) high pH and poor nutrient availability lead to fungal-plant associations that help deliver fitness benefits; and (c) novel fungi can be identified for their use in improving plant growth, and alleviate stress in diverse crops.

**Results:**

The study revealed that habitats with high pH and low nutrient availability have higher fungal diversity, with more rare fungi isolated from locations with chalky soil. Plants from location G were the healthiest even though soil from this location was the poorest in nutrients. Study of environmental gradients, especially extreme habitat types, may help understand the root zone fungal diversity of different functional classes. Two small *in vitro* pilot studies conducted with two isolates showed that endophytic fungi from suboptimal habitats can promote plant growth and fitness benefits in selected plant groups.

**Discussion:**

Targeting native plants and crop wild relatives for research offers opportunities to unearth diverse functional groups of root-derived endophytic fungi that are beneficial for crops.

## Introduction

Endophytes are ubiquitous asymptomatic microorganisms that live within plants in their natural habitats and are often symbiotic, which can have significant impacts on plant communities. The distribution of endophytes across environmental gradients reveals unique associations between fungi and plants (Redman & Rodriguez, 2007). Lau & Lennon (2012) propose that, when plants are faced with environmental change, they may benefit from association with diverse soil microbes that respond rapidly to such changes. These co-habiting belowground microbial communities could help maintain plant fitness by adapting to stressors associated with global climate change. Steps have been taken in understanding the relationships between endophytes and their hosts in a range of circumstances to study both biotic and abiotic stresses (Malinowski & Belesky, 2000; Bultman & Bell, 2003; Rodriguez & Redman, 2008; Oelmuller et al., 2009; Larriba et al., 2015) and these results underpin a framework for research to understand unique plant–fungal interactions and crop development strategies for the future in an age of climate change.

Crop wild relatives (CWRs) have provided breeders with genes for pest and disease resistance, abiotic stress tolerance, and quality traits in an ever-increasing number of food crops (Xiao et al., 1996; Hajjar & Hodgkin, 2007; McCouch et al., 2007; Khoury, Laliberté & Guarino, 2010). CWRs and other plants from suboptimal environments are also a source of genetic traits that can help productivity in challenging conditions (Mickelbart, Hasegawa & Bailey-Serres, 2015). Breeding of field crops for drought tolerance can be realized by using CWRs as discussed in tomato (Schauer, Zamir & Fernie, 2005), wheat (Nevo, 2007; Witcombe et al., 2008) and barley (Nevo, 2007; Lakew et al., 2011). Selection of superior plant genotypes and optimizing growing conditions to achieve the desired performance of crops is the conventional system currently used (Hale, Broders & Iriarte, 2014). They suggest that modifying the plant-associated microbiome may be an intriguing complementary strategy for crop improvement. Applying this to develop better crops using crop wild relatives (CWRs) and their microbiomes offers new possibilities.

On the other hand, the number of studies reported on asymptomatic fungi that are associated with the root zone of CWRs is still very few. Murphy, Doohan & Hodkinson (2015) found that novel, horizontally transmitted endophytes from a wild relative of barley may help the crop to grow successfully in nutrient-poor soil, which will help reduce fertilizer inputs while maintaining acceptable yields. The findings of this line of research may help to grow field crops in a more sustainable, cost-effective and environmentally friendly way (Murphy, Doohan & Hodkinson, 2015). As Hale, Broders & Iriarte (2014) suggested, in the same way that diversity loss was caused in crop species by domestication due to genetic bottlenecks, the process of migration may have resulted in loss of associated microbial diversity due to the physical dislocation of host plants from their co-evolved microorganisms.

Comby et al. (2016) found that, for both bacterial and fungal endophytes, there were strong spatial and temporal variations, and emphasized the importance of the establishment of a collection of cultivable endophytes that can be evaluated for their beneficial effects upon crops. Non-pathogenic endophytes might be of interest in the search for plant growth promoters or biological control agents (Alabouvette, Olivain & Steinberg, 2006; Berg et al., 2016). Two of the most important abiotic stressors are drought and salinity. In a world of changing climate, the importance of increasing the tolerance of crops to drought and salinity is critical (Tester & Langridge, 2010). Our current study was carried out to see whether suboptimal soil characteristics lead to environment-specific plant–fungal associations that can confer fitness benefits and improve plant performance.

We studied the distribution of fungal endophytes in one of the CWRs of strawberry, *Fragaria vesca*, across an environmental gradient within 1.6 km^2^ of protected land in Chafford Hundred, south-east England, managed by the Essex Wildlife Trust. The main objectives of this study included:

• Identification by ITS sequencing of cultivable endophytic fungi from roots of *Fragaria vesca*, on a spatial scale within a small geographical area of a protected woodland;
• Characterization of soil from seven collection locations for their key mineral element composition, pH and humus level;
• Diversity analysis of fungi in different soil types with regards to ubiquity and rareness;
• Assessment of wild *Fragaria vesca* plant characteristics to determine the influence of endophytes;
• Small pilot studies to understand the effect of two endophytic fungi on functional traits on fast-growing model species (selected cereal, legume and brassica).

## Materials & Methods

### Collection of material

All study materials were collected at Chafford Gorges Nature Park (CGNP), in Essex, UK. Since the plant materials were collected from areas that lie outside the Site of Special Scientific Interest (SSSI), a permit was not necessary. *Fragaria vesca* plant materials, therefore, were collected from areas outside SSSI with permission from CGNP.

**Figure 1:**
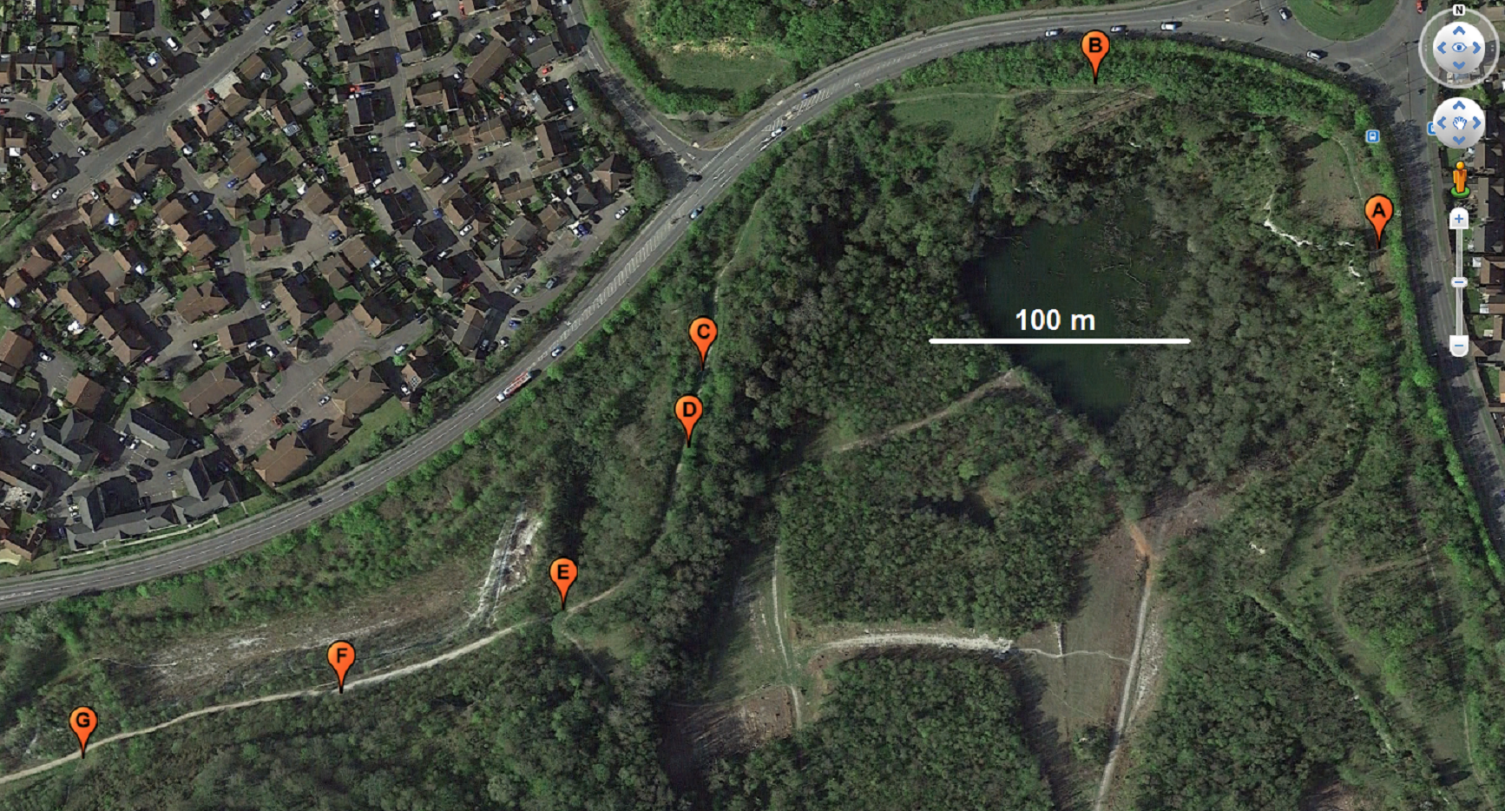
Locations (A–G) from where *Fragaria vesca* plant and soil samples were collected for the study. (Map data © 2016 Google).

Wild strawberry, *Fragaria vesca*, can be found in some areas of the Nature Park, mainly at the edges of wooded areas where there is intermediate shade and vegetation cover. Seven collection locations (A–G, Table 1 and Fig. 1), along an unpaved path were chosen for study. Locations A and G were the furthest from each other, with a straight-line distance of 530 m. In June 2015, two plants (1 and 2) were collected from each location. Three soil samples (*ca.* 300 g per sample from depth of 0 to 5 cm from the soil surface) were collected from each location in February 2016.

**Table 1:** The description of the collection locations. The locations and elevation were determined using a combination of GPS-enabled devices and Google Earth images.

Location	Straight line distance from previous location (m)	Elevation (m)	Description
A	n/a	20	Along path; 3–4 m from hedgerow; vegetation regularly cut to maintain hedgerow boundary
B	123	20	Along path; 1–2 m from hedgerow; vegetation regularly cut to maintain hedgerow boundary
C	183	17	Close to edge of path; 1 m from hedgerow; vegetation regularly cut to maintain hedgerow boundary
D	30	14	Close to edge of path; 1–2 m from hedgerow; vegetation regularly cut to maintain hedgerow boundary
E	81	12	Close to edge of path; 1 m from hedgerow; vegetation cut less frequently
F	88	13	3–4 m from path; 4-5 m from, and shaded by, chalk cliff; under trees (birch); ground covered in leaf litter
G	91	12	5–8 m from chalk cliff; elevated from path level; ground covered in moss, with little topsoil over chalk rock; shaded under shrubby trees; roots white from adhering chalk

### Fungus isolation and identification

The three longest roots of each plant were surface sterilized in 0.5% (w/v) sodium dichloroisocyanurate, with 0.1% (v/v) Tween-20 as surfactant, for 15 min and rinsed in sterile water. The roots were put onto fungus isolation medium (FIM; Mitchell, 1989) containing streptomycin sulphate (Clements & Ellyard, 1979) in Petri plates sealed with Parafilm, and incubated at 18 °C. Tips of fungal hyphae emerging from the roots were isolated and cultured on fresh FIM in sealed 5 cm Petri plates.

For taxonomic identification of each fungal isolate, the internal transcribed spacer region of the rRNA gene (ITS) was sequenced, essentially as described in Yokoya et al. (2015). Briefly, DNA was isolated from a sample of mycelium and the ITS was amplified by PCR using primers ITS1 and ITS4 (White et al., 1990). The amplified DNA samples were sequenced by Sanger sequencing with both forward and reverse primers using Applied Biosystems 3,730 × l DNA Analyzer (Life Technologies, Carlsbad, CA, USA). All sequence analyses were performed using Geneious^®^ software package (Biomatters, Auckland, New Zealand). The forward and reverse sequences were checked for accuracy and consensus. Isolates with sequences that matched by 99% or more were grouped in to the same operational taxonomic units (OTUs), except for basidiomycete fungi for which a conservative similarity threshold of 97% was used, to take into account the higher infraspecific variability within the ITS region in this group, including heterozygosity and single nucleotide indels (Hughes, Petersen & Lickey, 2009). Representative sequences of each OTU were used to query the GenBank database using BLAST (National Center for Biotechnology Information, Bethesda, MD, USA) for a tentative identity based on named database entries with the most similar sequences.

### Soil analysis

The soil samples were analysed using LaMotte STH Series Combination Soil Testing Outfit according to the manufacturer’s instructions (LaMotte Chemical Products 1967; LaMotte, Maryland, USA). Analyses were carried out for pH, ammonium, nitrite, nitrate, humus, calcium and P (using the alternative phosphorus detection kit for alkaline soil).

### Plant morphometry

Number of leaves, petiole length, leaf length and breadth, and longest root measurements were recorded from the same two plants collected from each location for fungus isolation. Only one plant collection was made so that it would not adversely affect the existing population, as the plants were from a conservation area within the wildlife site.

### *In vitro* growth for biomass and salt tolerance studies

Detailed studies on the effect of selected endophytes of *F. vesca* on plant growth and salt tolerance *in vitro* were conducted on fast growing cereal, brassica and legume seedlings. The same endophytes were also used to see their effect on cultivated strawberry, *Fragaria* × *ananassa* ‘Anablanca’; however, it was not used for further study due to its slow growth *in vitro*. Two week-old *in vitro* seedlings of rye (*Secale cereale*) and common vetch (*Vicia sativa*) germinated on agar-solidified (8 g l-1), sucrose-free half-strength Murashige and Skoog salts and vitamins (Murashige & Skoog, 1962) were transferred to Magenta^®^ jars (five replicate seedlings per treatment group), with B caps which allows passive air exchange, (Sigma Chemicals, Poole, UK) with 40 ml of the same medium. As F and G locations were different from the others in terms of fungal diversity and rarity, two rare endophytes from these locations were selected for further study. Hyphae from isolates of *Humicola* sp.-like F2A(13) and *Volutella rosea*-like G3B(10) were taken from actively growing cultures in 3 mm cubes of agar and transferred to the surface of the plant culture medium, being placed at 1 cm from the seedling. The control treatment remained uninoculated. Radish (*Raphanus sativus*) was selected for the salt tolerance study (nine replicate seedlings, three in three jars, per treatment group), under similar culture conditions but with the addition of 150 mM salt (NaCl) in the medium. All cultures were incubated at 24 ± 2 °C for eight weeks for biomass and 12 weeks for salt tolerance studies on a 16/8 photoperiod under cool white fluorescent tubes at 50 µmol photons m-2 s-1. Data collected from rye and common vetch include fresh and dry weights of both shoot and root systems. Shoot height, number of root branches, and the amount of medium left in the culture vessel were collected for the salt tolerance study in radish.

For the *in vitro* pilot studies, the significance of differences between means was assessed first by one-way ANOVA using the proprietary function in Microsoft^®^ Excel^®^ 2013. Post hoc pairwise comparisons with the control were made using the Games-Howell test (Games & Howell, 1976) and using tables for critical values of q for the Studentized Range distribution at *α* = 0.05 and 0.01 (Kokoska & Nevison, 1989). Due to mortality of some of the radish seedlings in the salt tolerance study, eight replicate seedlings of the control and *Volutella rosea*-like G3B(10) treatments, and seven replicate seedlings of the *Humicola* sp.-like F2A(13) treatments were considered for the statistical analysis.

### Statistical analysis

Statistical analyses of fungal diversity were performed with R version 3.2.4 (R core Team, 2016). Multivariate analyses of fungal distribution and four environmental gradients were made using non-metric multidimensional scaling (NMDS), with *metaMDS* and *envfit* functions in the R package vegan (Oksanen, 2015). Forty two root samples collected from 7 sites of CGNP (6 samples from each site) were subject to NMDS analysis, in which the species data set with record of 61 fungi (OTUs) observed across those 42 root objects. Another data set with measurement of Ca, Humus, P and pH levels across those 42 objects was used to fit environmental vectors onto ordination with *envfit* function. The environmental vector arrow points to the direction of the gradient, and the length of the arrow is proportional to the strength of the gradient. The significance of fitted four environmental vectors (*p* value) is assessed using permutation of environmental variables. The goodness of fit statistic is squared correlation coefficient (*r*^2^).

For the *in vitro* pilot study, the significance of differences between means was assessed first by one-way ANOVA, and *post hoc* pairwise comparisons with the control were made using the Games-Howell test.

## Results

### Fungi isolation and distribution

Fungal growth was observed after 24 h in root sections. Emerging hyphae were isolated during the following 10 days and individually cultured. Density of fungal colonization on the roots varied depending on different groups of fungi. Of 457 isolates, full-length or near full-length ITS sequences that were sufficiently reliable for subsequent analysis were obtained from a total of 415 isolates; 42 isolates with poor sequencing results were not considered in the subsequent analysis. The sequences obtained were classified into 61 operational taxonomic units (OTUs) based on a sequence similarity of 99%, except for basidiomycete fungi where a threshold of 97% was used to take into account the greater ITS variability found in this taxon (Hughes, Petersen & Lickey, 2009). Closest matches in BLAST search results against the GenBank database were used to assign most-closely related taxa. A fungal isolate was assigned to the most related species if the pairwise match of 99–100% was made to a named GenBank entry, with no other close matches of a different species name. Otherwise, tentative identifications were made to genus, family or order.

Of the 61 OTUs, 12 were defined as ‘common’ fungi, for being present in three or more locations. Representatives of common fungi were present in all seven locations (Table 2). However, locations F and G had a greater number of ‘rare’ fungi, defined here as OTUs that were found in only one or two locations.

**Table 2:** Number of rare and common OTUs at each location.

Location	Common^a^	Rare^b,c^	Rare, shared locations
A	6	6 (3)	1B; 1C; 1D
B	9	5 (4)	1A
C	7	6 (3)	1A; 1D; 1F
D	6	5 (2)	1A; 1C;1G
E	7	7 (6)	1G
F	7	11 (10)	1C
G	7	16 (14)	1D; 1E

**Notes.**

a Common fungi were defined as being present at three or more locations.

b Rare fungi were defined as being present at only one or two locations.

c Number in parentheses indicate unique OTUs, found only in the corresponding location.

**Figure 2:**
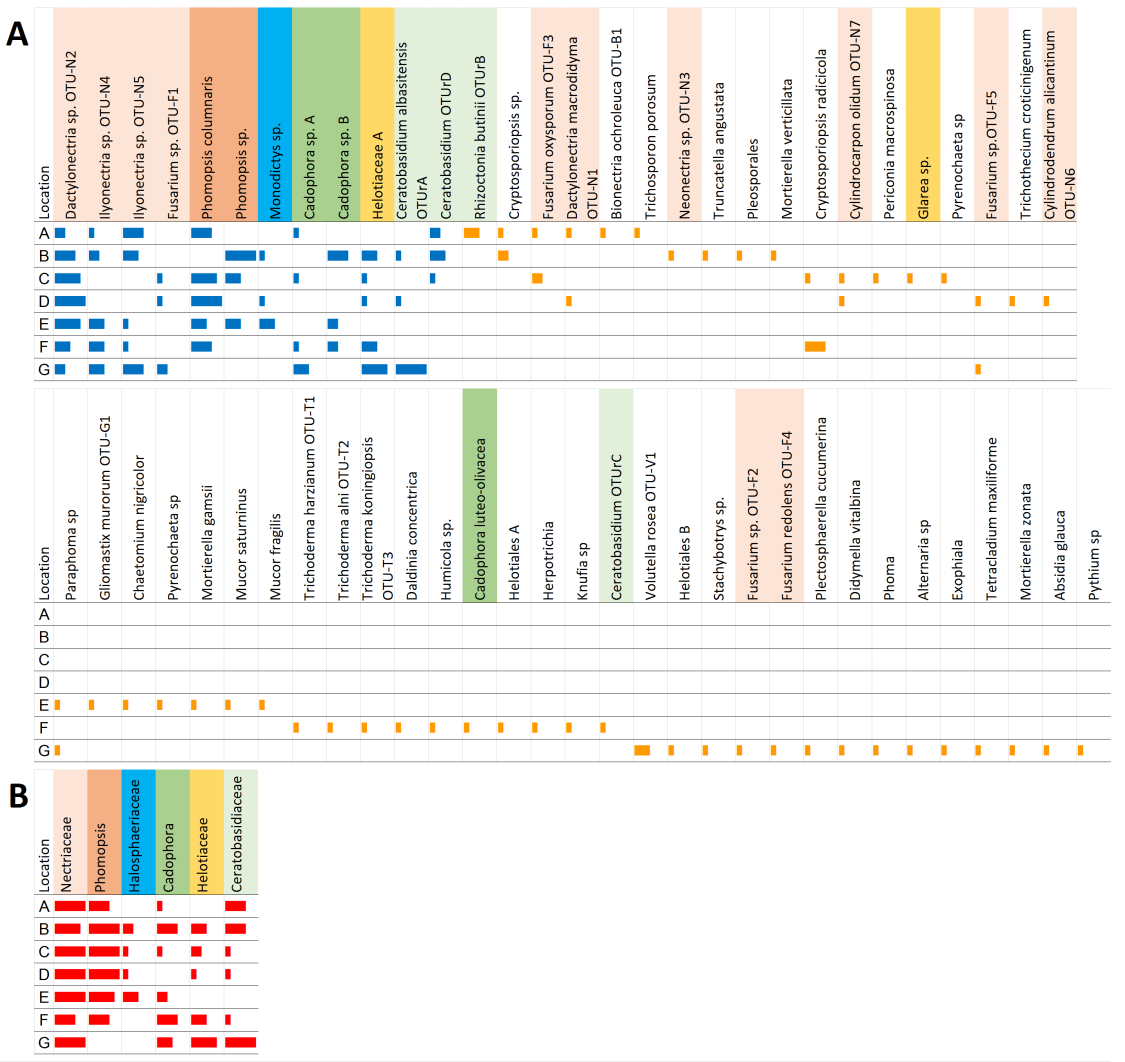
Abundance of the 12 common (blue bars) and 49 rare (orange bars) OTUs at each location. (A) Three roots from each of two plants were cultured from each location; with six roots per location, the isolation of the same OTU from all six roots from the same location scored a maximum score of 6 for abundance (fully horizontally extended bar). (B) Distribution of the OTUs in each location after their grouping into families (red bars).

Only one (*Dactylonectria* sp.-like OTU-N2) of the 12 common OTUs were found in all seven locations. The distribution was not uniform, and an abundance plot (Fig. 2A) showed that, for most OTUs, the abundance showed either a ‘peak’ or a ‘trough’ around locations C or D, as well as at G. The uneven distribution was also apparent when the abundance of the common fungi were combined into taxonomic families (Fig. 2B). It can also be seen that within the families with multiple OTUs, such as Neonectriaceae, Diaporthaceae, Helotiales *incertae sedis* containing *Cadophora* sp., or Ceratobasidiaceae, the different OTUs have different, sometimes contrasting, distribution patterns, such that some OTUs are most abundant at locations where the other OTUs are absent (Fig. 2).

An abundance plot of the rare OTUs (Fig. 2A) showed that F and G had many more rare fungi compared to the other locations. Although defined as being from one or two locations, rare fungi were often found only from one location (Table 2); furthermore, they were typically only from one root of the six from a given location, being consequently defined as ‘singletons,’ i.e., OTUs represented only by a single isolate. Among the rare OTUs, the maximum abundance score in one location was 4.

**Figure 3:**
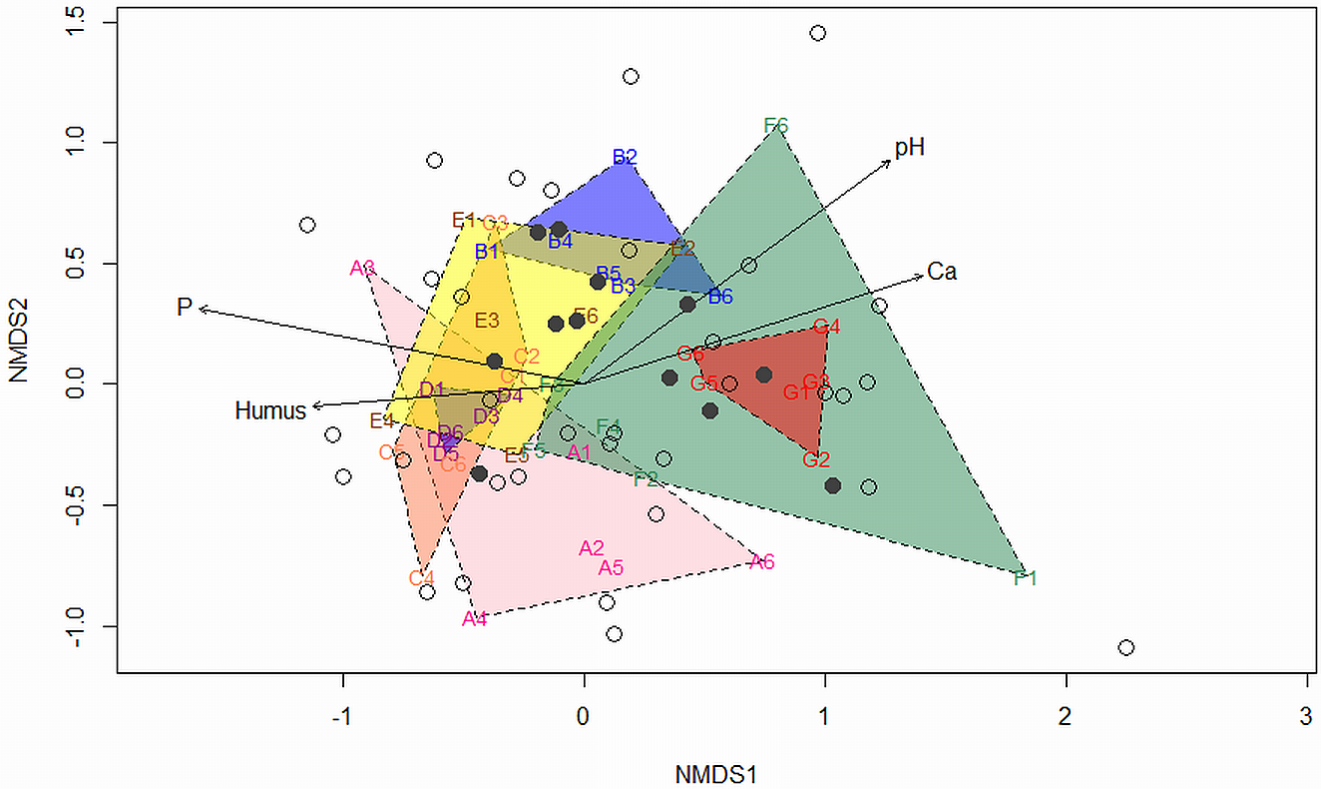
Non-metric multidimensional scaling (NMDS) plot showing diversity of root-derived endophytic fungi among six roots from each of seven locations (A–G) along environmental gradients (levels of Ca, humus, P and pH). Filled grey circles indicate common endophytes; empty grey circles indicate rare endophytes. The four fitted environmental vectors showed correlations on the ordination plot with significance (Ca, P and pH: *p* < 0.01; humus: *p* < 0.05).

The NMDS plot of six roots collected from each of seven locations, showed that samples collected in locations C and D were most separated from G along the horizontal axis (Fig. 3). Vectors of the four environmental variables also aligned roughly parallel to the NMDS1 axis. Overall, the 12 common OTUs (shown as Filled Grey Circles) were closer to the average soil condition (the centre), relative to the rare OTUs (Empty Grey Circles).

### Soil analysis

Soil samples from locations A, B and C had a dense, clay-rich soil, but D showed very little clay. Soil from location E was also loamy, while soil samples from F and G were noticeably chalky-white. Soil acidity varied, generally from near neutral pH at the start (A) to high pH at the end of the path (G). Exposed chalk landscape and white chalk in the soil can be seen, particularly at locations E, F and G, which explains the relatively high pH and calcium concentration. Measurable nitrogen was low, with low ammonia and nitrite; nitrate was highest at B, C and D (15 lb acre^−1^). *P* levels mirrored this pattern, which was also low at A and at F and G (ca. 10 lb acre^−1^), and highest at C, D and E (75 lb acre^−1^). Humus content was the highest at B, C and D, and low at E and G. Calcium was very high throughout but, as expected, it was the highest at F and G (4.4%) and lowest at A (0.35%).

Overall, the results showed a gradient with increasing pH and calcium concentration from A to G, and a peak of humus, P and nitrate at C and D, indicating greatest organic decomposition in these locations in the middle of the gradient.

### Plant morphometry

**Figure 4:**
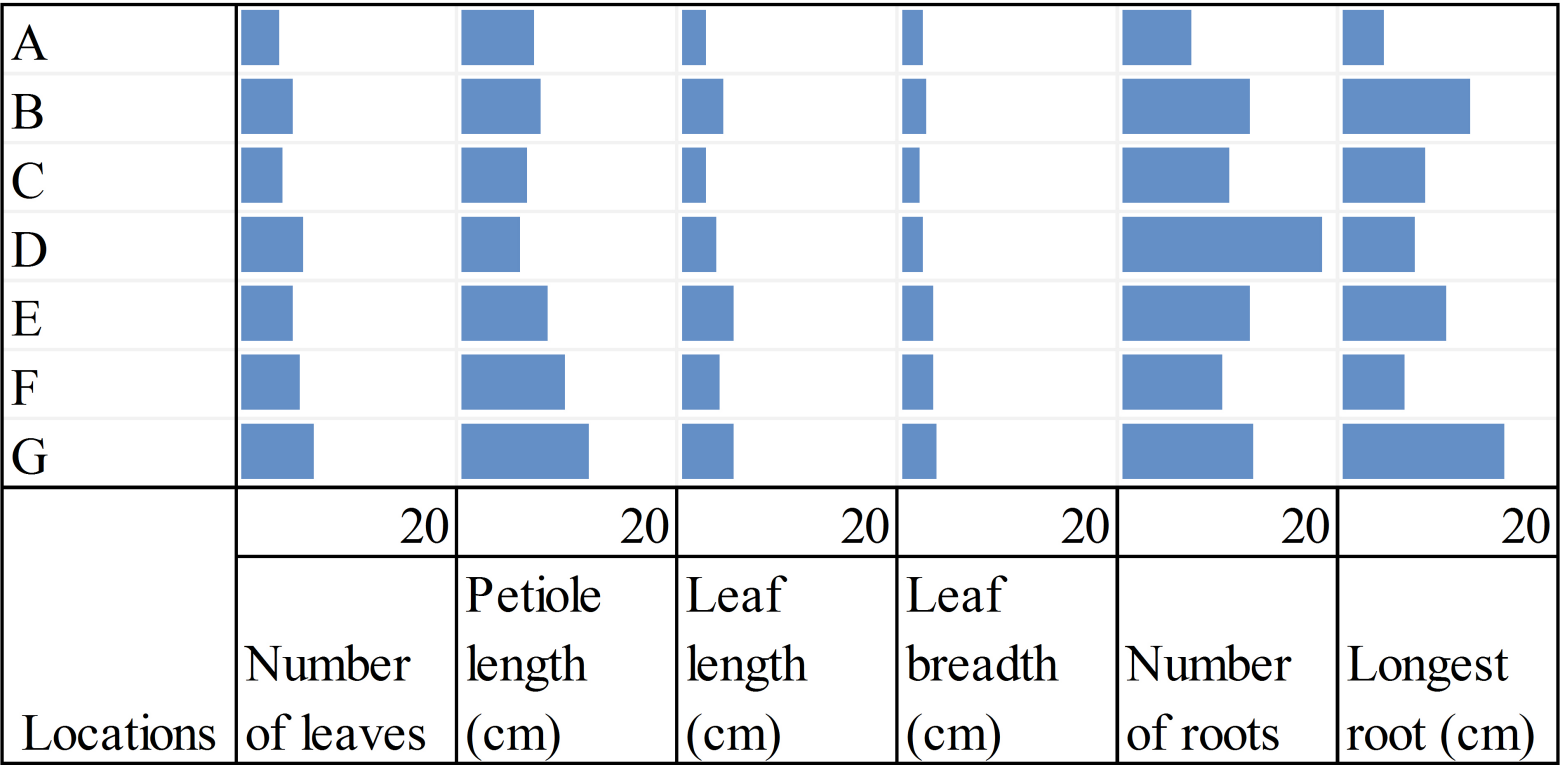
Shoot and root measurements of wild strawberry, *Fragaria vesca*, from seven locations (A–G). Data represents means for each characteristic from two plants from each location.

Total number of leaves, petiole length, leaf area, number of roots and longest root were recorded from collected plants (Fig. 4). The smallest plants were found in location A where soil pH was 7.9 and with low P, calcium, and no detectable ammonium. Location G, on the other hand, had plants with the highest number of leaves, the longest petioles, widest leaves and the longest roots, and ranked second for leaf length and number of roots.

**Figure 5:**
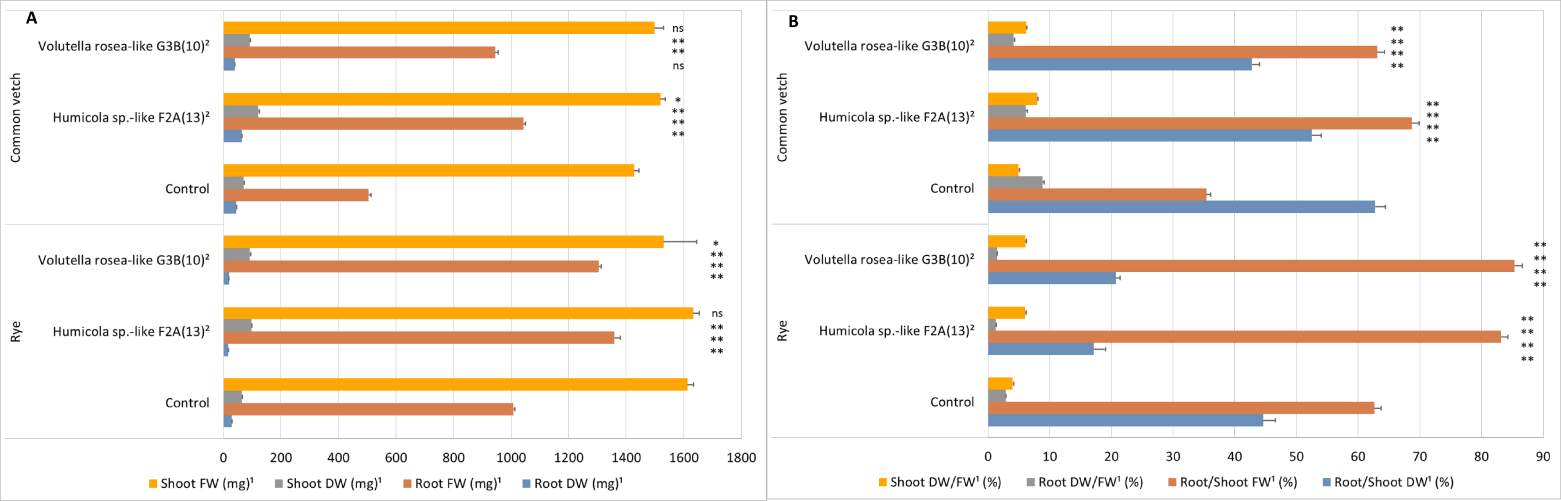
Effect of endophytes *Humicola sp*.-like F2A(13) and *Volutella rosea*-like G3B(10) on shoot and root weights of rye and common vetch. (A) Fresh weight (FW) and dry weight (DW) of shoots and roots. (B) DW/FW ratios and root/shoot weight ratios. ^1^Mean ± standard errors are shown (*n* = 5 for each treatment group). ^2^Statistically significant differences are indicated (ns: not significant; ^∗^: significant at 0.01 ≤ *p* < 0.05; ^∗∗^ significant at *p* < 0.01) as determined by comparison of treatment to control using the Games–Howell test.

### *In vitro* endophytic mutualism pilot study

**Figure 6:**
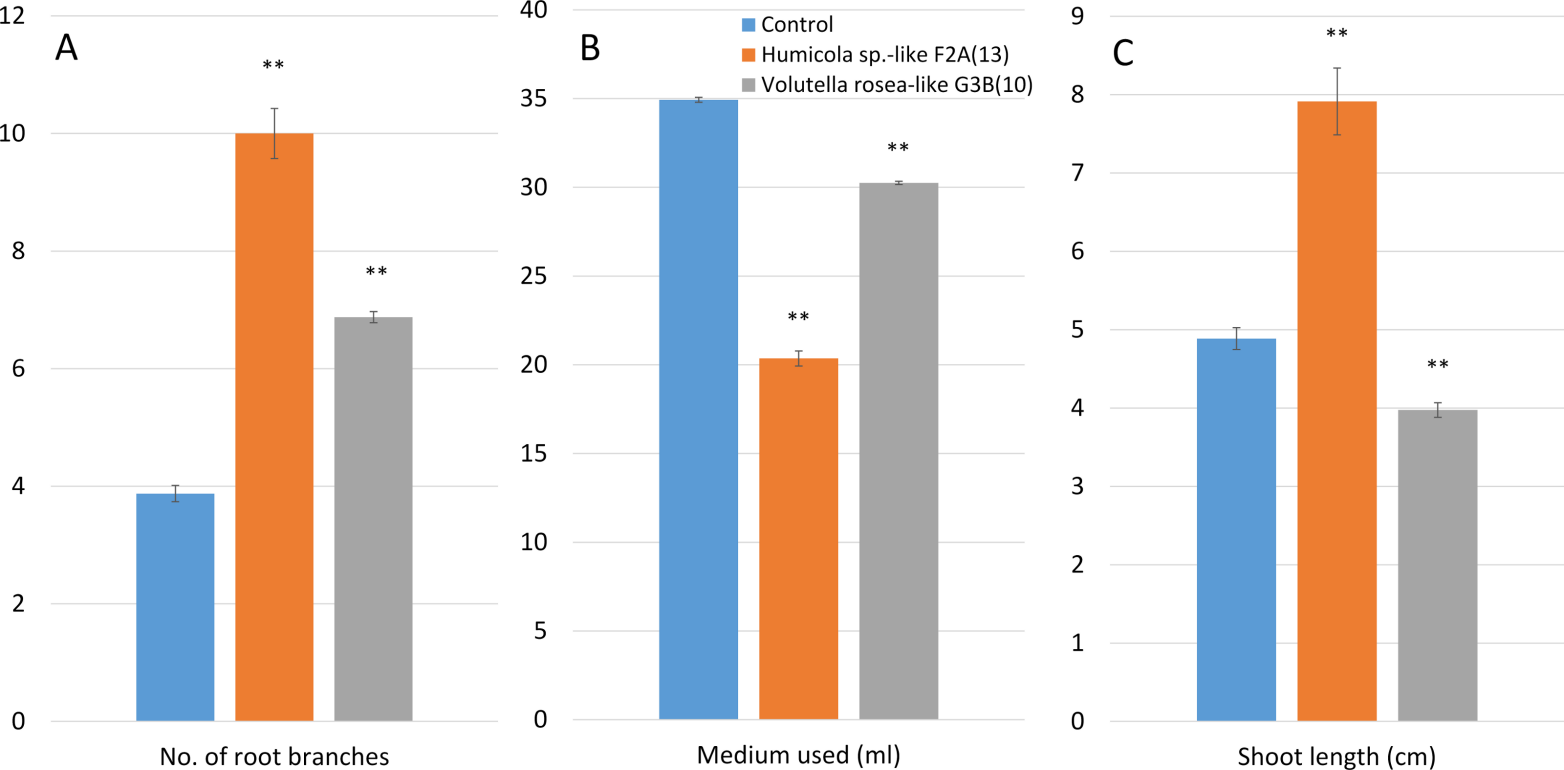
Effect of *Humicola sp*.-like F2A(13) and *Volutella rosea*-like G3B(10) on radish cultures that have been co-cultured with one of the endophytes for 12 weeks *in vitro* on sucrose-free medium. Mean ± standard errors are shown (*n* = 7 for *Humicola sp*.-like F2A(13), *n* = 8 for control and *Volutella rosea*-like G3B(10)) for (A) number of root branches that were longer than 10 mm, the (B) volume of medium used from a starting volume of 40 ml, and (C) shoot length. Statistically significant differences are indicated (∗∗); all treatments differed from control at *p* < 0.01 as determined by comparison of treatment to control using the Games–Howell test.

(a) Biomass
  *In vitro* cultures of rye and common vetch seedlings, grown on sucrose-free half-strength Murashige & Skoog (1962) medium, were used for studies on endophytic mutualism. In both rye and common vetch, there were statistical differences with endophyte treatments for each variable measured, as determined by ANOVA, compared to endophyte-free controls. However, for both plant species, only one of the two endophytes tested resulted in a significant difference in shoot fresh weight from that of control plants; in common vetch, shoot fresh weight increased with *Humicola* sp.-like F2A(13), but decreased in rye with *V. rosea*-like G3B(10). On the other hand, the shoot dry weight of both rye and common vetch was significantly higher compared to the control by both *Humicola* sp.-like F2A(13) and *V. rosea*-like G3B(10) (Fig. 5A). Both endophytes also resulted in a statistically significant increase in the fresh weight of roots when co-cultured with rye and common vetch. However, root dry weight was unchanged or significantly reduced when co-cultured with either endophyte, except for common vetch with *Humicola* sp.-like F2A(13) where it was increased after co-culture.
  In both rye and common vetch, root to shoot ratio by fresh weight was increased when plants were grown with endophytes, but the root to shoot ratio by dry weight was decreased (Fig. 5B). In both rye and common vetch, dry weight relative to fresh weight of shoots increased when plants were grown with endophytes, but the dry weight relative to fresh weight of roots decreased with co-cultured fungi compared to controls (Fig. 5B).
(b) Salt tolerance
  In our small pilot study, number of root branches was significantly increased by the presence of either of the two endophytes tested (Fig. 6A). Additionally, the amount of water used by radish plants was significantly reduced by the presence of both *Humicola* sp.-like F2A(13) and *V. rosea*-like G3B(10), based on the volume of medium used (Fig. 6B). Over 12 weeks 20.4 ml and 30.3 ml of medium was used in cultures with *Humicola sp.*-like F2A(13) and *V. rosea*-like G3B(10), respectively, while the control plants used 34.9 of the original 40 ml during this period, which eventually led to the death of plants due to desiccation and, presumably, from excessively increased NaCl concentration in the medium. Shoot length was significantly increased with *Humicola sp.*-like F2A(13) but was significantly reduced with *V. rosea*-like G3B(10) (Fig. 6C).

## Discussion

### Ubiquity against rarity along environmental gradients

*Fragaria vesca* from seven collection locations yielded at least 61 different endophytes, demonstrating the ability of this plant species to associate with a wide range of fungal groups. The differences in fungi recovered from different locations reinforce the idea that the interactions in plant-endophyte associations vary with environmental conditions (Wali et al., 2009), even between habitats that are very proximal geographically (few hundred meters). The locations F and G were notable in their greater root fungus diversity. The NMDS plot (Fig. 3) showed that location G was well separated, particularly from locations C and D, demonstrating their difference to each other in terms of associated endophytes and underlying environmental gradients. The NMDS with overlaid environmental vectors correlates endophytes at location G with higher pH and calcium levels, and low levels of phosphate and humus. This suggests that the 16 rare fungi observed only in samples from location G (Table 2) may be key beneficial endophytes that help the host plant to tolerate environmental stresses. *Trichoderma* isolates were found only in location F which may substantiate the notion that root colonization by *Trichoderma* spp. can confer resistance to abiotic stresses, as well as improve the uptake and use of nutrients (Harman et al., 2004). These two locations also had relatively little small vegetation, both in quantity and diversity, which may also have an influence upon fungal diversity. The more common fungi may be associated with, and dependent on, specific non-strawberry species, which may be uncommon or absent at location G. With reduced dominance of common fungi, other endophytes, which are adapted to different environments, may then be able to colonize the *F. vesca* plants.

### Plant morphology, P-limiting soil and fungal diversity

*Phomopsis columnaris*, *Ilyonectria robusta* and *Dactylonectria* sp. were more abundant in locations that had relatively nutrient-rich soil. In suboptimal environments as location G, it would be beneficial for plants to be able to have a mutualistic relationship with microbes that can release the precipitated P to make it available to the plant. Fungal endophytes have been shown to increase plant P uptake (Behie & Bidochka, 2014), and Hartmann et al. (2009) showed that roots exude a variety of compounds giving the plants a level of selectivity over potentially endophytic microbes. The potential of microorganisms for synthesis and release of pathogen-suppressing metabolites have been shown in previous *in vitro* studies (Vassilev, Vassileva & Nikolaeva, 2006), strongly supporting manipulation of soil microbiota for agricultural applications. This could explain the improved fitness in terms of increased size of plants at location G despite its poor-nutrient soil (Table 3 and Fig. 4).

**Table 3:** Soil analysis data from each location.

Location	*p*H^a^	NH${}_{4}^{+}$ (ppm)^b^	NO${}_{2}^{-}$ (ppm)^b^	NO${}_{3}^{-}$ (lb acre^−1^)^b^	P (lb acre^−1^)^b^	Ca^2+^ (ppm)^a^	Humus (%)^a^
A	7.9 ± 0.1	0	<1	<10	<10	3,500	9.1 ± 1.4
B	8.4 ± 0.1	<5	<1	15	25	17,500	11.8 ± 0.5
C	7.9 ± 0.0	<5	<1	15	75	17,500	11.5 ± 2.2
D	8.3 ± 0.1	<5	<1	15	75	17,500	11.7 ± 2.0
E	8.6 ± 0.0	<5	<1	≤10	75	25,000	3.9 ± 1.6
F	8.6 ± 0.1	<5	<1	<10	10	43,750	8.4 ± 2.8
G	8.9 ± 0.2	<5	<1	<10	<10	43,750	2.7 ± 1.2

**Notes.**

a Average shows mean ± SE of three samples.

b Average shows mode of three samples.

The significance of soil pH was found to be critical for the presence of a specialised sebacinalean endophyte (López-García, 2016) while Bååth & Anderson (2003) observed fungal biomass increased with increasing soil pH. Cultivated strawberry often suffer from chlorosis when grown in high pH soil (Yao & Guldan, 2015), but wild strawberry is often found on chalky land (Grubb, Green & Merrifield, 1969). It is possible that *Fragaria vesca* has an established symbiosis with endophytes that help with nutrient availability in high pH conditions. The pH gradient observed from locations A to G suggests a longer period of topsoil accumulation at A. At location G the more recent exposure of chalk bedrock is still evident, including visible chalk in the substrate. LaMotte Chemical Products (1967) states that 75 lb acre^−1^ of readily available phosphorus is the minimum required level for agricultural use, and 200 to 300 lb acre^−1^ or more is desirable. Location G had a very low P content (<10 lb acre^−1^) compared to other locations (Table 3). It is therefore noteworthy that the *F. vesca* plants at G were apparently as healthy as at any of the other collection locations, with the highest amount of roots and the longest root. Vigorous growth of long and dense roots is an important prerequisite for efficient acquisition of macro and micro nutrients (Wang et al., 2016) and root hair length is critical for the acquisition of limited nutrients such as P (Brown et al., 2012). Valentinuzzi et al. (2015) reported that P and iron deficiency had a positive effect on the nutritional content of strawberry fruits without negative effects on fruit yield and quality. The above finding suggest that it could be possible to grow strawberry in nutrient-limited soils enriched with endophytes to improve the resilience and qualitative aspects of the crop.

### Potentially beneficial fungal endophytes among ‘rare’ fungi

Majority of ‘rare’ fungi identified from both locations F and G are of interest for further detailed studies. The isolate *Humicola* sp.-like F2A(13), recovered from location F, showed to be most closely related to *Humicola* spp. (Sordariales, Chaetomiaceae), a fungal genus found to confer benefits to plants (Lang et al., 2012; Radhakrishnan et al., 2015). Some *Humicola* species are asymptomatic root fungi such as *H. fuscoatra* in tomato roots (Menzies et al., 1998), while in other cases they showed promise to be useful in the biological control of plant pathogens (Ko et al., 2011; Piper & Millson, 2012; Wicklow et al., 1998; Wicklow, Jordan & Gloer, 2009). Yang et al. (2014) also discussed its role as a biocontrol agent against *Phytophthora* spp. Isolates, including *Volutella rosea*-like G3B(10), with 100% ITS sequence match to *Volutella rosea* were found only in location G. *Volutella* is able to solubilize and mineralize P from inorganic and organic pools of total soil P to make them available to plant roots (Vassilev, Vassileva & Nikolaeva, 2006; Richardson, 2001). Carnivorous plants that live in nutrient-poor and stressful environments were found to benefit from the presence of *Volutella* (Quilliam & Jones, 2012). Another endophyte, most closely related to *Paraphoma* sp. (Pleosporales, Pleosporaceae) was found in both F and G locations. Zhang et al. (2012) found that a specific strain of *Paraphoma* improved the biomass of its host plant. Endophytes related to other fungal genera of interest include *Trichoderma*, *Knufia* and *Exophiala*. *Trichoderma* can stimulate plant growth and defence responses have already been widely used for biological control of plant diseases (e.g., Harman et al., 2004; Druzhinina et al., 2011); *Knufia* and *Exophiala* species have been found to be highly stress-tolerant fungi in hot and arid environments (Zakharova et al., 2014).

### Improvement of plant growth in culture by *Humicola***sp.-like F2A(13) and***Volutella rosea* -like G3B(10)

Biomass of rye and common vetch was influenced when plants were grown with both *Humicola* sp.-like F2A(13) and *V. rosea*-like G3B(10) isolates. As a direct impact of endophyte inoculation, the fresh weight root/shoot ratio of both rye and common vetch was increased (Fig. 5). Wali et al. (2009) and Ding, Kupper & McNear Jr (2015) also found higher root/ shoot ratios in different species of *Festuca* with endophytes compared to endophyte-free plants. On the other hand, the root/shoot ratio of dry weight in both plant species were less in the presence of endophytes. Although the root dry weight/fresh weight percentage was significantly lower in endophytic treatments, this was probably so due to longer and thinner roots in rye and greater branching in common vetch plants. These conditions, on the other hand, may have effectively improved the surface area when compared to controls, thereby allowing a relative increase in nutrient uptake.

Association with beneficial fungi stimulates increased growth of plants, presumably by increasing nutrient acquisition of the plant. In our study, both endophytic isolates tested *in vitro* promoted increase in root fresh weight. A variety of factors may be involved in the stimulation of plant growth when P availability is low, one of the critical variables being root architecture (Burridge & Jochua, 2016). Endophytic fungi may effectively improve P absorption by altering root architecture (Ding, Kupper & McNear Jr, 2015). The significant increase in shoot dry weight and in root/shoot fresh weight percentage in both rye and common vetch have apparently highlighted this aspect (Fig. 5). The increase in the ratio of shoot dry weight/fresh weight percentage also suggests the improved quality of the shoot system in both plants. Interaction with fungi may be one of the reasons why plants from location G had longer roots in the wild. It seems reasonable to suggest that plants with long and dense root systems can compete more effectively for nutrients both *in situ* and *in vitro* as found in this study.

### Effect of *Humicola* sp.-like F2A(13) endophytic isolate on salt tolerance

Plants from the Brassicaceae family are known to have no mycorrhizal association, so it was envisaged that other root endophytes, including those derived from other species, might have particular significance for this family of plants (Card et al., 2015). In their review of Brassicaceae endophytes, Card et al. (2015) noted that endophytes recovered from crop wild relatives (CWRs) of brassica are beneficial for different characteristics related to growth and tolerance to stresses in plants. In this work, the brassica plant radish was studied for salt tolerance and water use efficiency under *in vitro* conditions using inoculation with the *Humicola* sp.-like F2A(13) and *V. rosea*-like G3B(10) endophytic isolates. Our results indicated that both endophytes helped radish to use water more efficiently. Since evaporation from the medium surface and transpiration through plant tissue should be the only routes of water loss from the medium (escaping through the Magenta^®^ vessel B caps which allow passive air exchange), the rate of transpiration was presumably influenced by the presence of fungi. Plants co-cultured with *Humicola* sp.-like F2A(13) had greater shoot length and might therefore be expected to lose more water through a greater plant surface area. Neverthless, these plants depleted the least amount of medium volume (Fig. 5). Radhakrishnan et al. (2015) found that a *Humicola* sp. isolate significantly increased shoot length and protein content in salt-stressed plants of soybean. In agreement with these data, our results suggest that both *Humicola* sp.-like F2A(13) and *V. rosea*-like G3B(10) isolates are promising fungi to be further explored in their potential ability to confer salt-stress tolerance. Research in using co-inoculation with multiple microorganisms rather than with a single inoculant can improve plant yield (O’Callaghan, 2016). We suggest that the use of other endophytes identified from F and G locations could also be explored further in terms of potential bioinoculants for different crops and model plant species, using *in vitro* and *ex vitro* systems, either individually or in combinations.

### Endophytes of CWRs along environmental gradients

Suboptimal environments offer opportunities to explore and understand the fungal diversity that could be useful for agricultural applications. For example, novel endophytes of plants growing in geothermal soils have been shown to confer high temperature tolerance to the host plants (Rodriguez & Redman, 2008; Zhou et al., 2015). The majority of the CWRs originate from diverse habitat types, including hotspots in the subtropical and tropical regions of the world (Castañeda-Álvarez et al., 2016). Studying CWRs and their root-derived and foliar endophytes along environmental gradients could help uncover a whole new array of beneficial fungi. Detailed studies on the benefit of fungi sourced from P-limited soil types (e.g., from F and G locations) are ongoing to understand whether selected endophytes can support better growth of plants on P-limited growing media.

## Conclusions

This study targeted a small geographical area and a CWR from a single plant taxon to understand the fungal endophytic diversity along environmental gradients. A greater number of ‘rare’ fungi was found in the location with high pH and poor nutrient availability. It has already been recognized that the plant microbiome should be considered in crop breeding strategies (Berg et al., 2016). Studying endophytes of CWRs will help understand the plant-fungus relationship and their impact on growth and stress tolerance in crop species, and further research will shed light on habitat adaptation and plant succession in marginal and challenging habitats from where many CWRs originated. As a result of domestication and migration, it is likely that present crop species have become devoid of beneficial microbes which are part of the rhizosphere of CWRs (Hale, Broders & Iriarte, 2014; Pérez-Jaramillo, Mendes & Raaijmakers, 2016). Using less resources for better crop production is a sustainable formula for future crop growing systems, and endophytes may play a significant role (Jones, 2013). Based on the preliminary results obtained from our findings, detailed studies are in progress to understand the potential beneficial effects of these fungi to plants. Endophytes from challenging environments could offer opportunities to develop smart solutions to combat both abiotic and biotic stressors in the age of climate change and continued human population growth.

##  Supplemental Information

10.7717/peerj.2860/supp-1 Data S1 Raw data for NMDs analysis

10.7717/peerj.2860/supp-2 Data S2 Raw data

10.7717/peerj.2860/supp-3 Table S1 GenBank sequence matches and accession codes of isolated OTUs

## Additional Information and Declarations

### Field Study Permissions

The following information was supplied relating to field study approvals (i.e., approving body and any reference numbers): Essex Wildlife Trust, UK.

### DNA Deposition

The following information was supplied regarding the deposition of DNA sequences: GenBank KX610376– KX610460.

### Data Availability

The following information was supplied regarding data availability: The raw data has been supplied as Supplemental File.

## References

[ref-1] Alabouvette C, Olivain C, Steinberg C (2006). Biological control of plant diseases: the European situation. European Journal of Plant Pathology.

[ref-2] Bååth E, Anderson TH (2003). Comparison of soil fungal/bacterial ratios in a pH gradient using physiological and PLFA-based techniques. Soil Biology and Biochemistry.

[ref-3] Behie SW, Bidochka MJ (2014). Nutrient transfer in plant–fungal symbioses. Trends in Plant Science.

[ref-4] Berg G, Rybakova D, Grube M, Koberl M (2016). The plant microbiome explored: implications for experimental botany. Journal of Experimental Botany.

[ref-5] Brown LK, George TS, Thompson JA, Wright G, Lyon J, Dupuy L, Hubbard SF, White PJ (2012). What are the implications of variation in root hair length on tolerance to phosphorus deficiency in combination with water stress in barley (*Hordeum vulgare*)?. Annals of Botany.

[ref-6] Bultman TL, Bell GD (2003). Interaction between fungal endophytes and environmental stressors influences plant resistance to insects. Oikos.

[ref-7] Burridge J, Jochua CN, Bucksch A, Lynch JP (2016). Legume shovelomics: high—throughput phenotyping of common bean (*Phaseolus vulgaris* L.) and cowpea (*Vigna unguiculata* subsp, *unguiculata*) root architecture in the field. Field Crops Research.

[ref-8] Card SD, Hume DE, Roodi D, McGill CR, Millner JP, Johnson RD (2015). Beneficial endophytic microorganisms of Brassica—A review. Biological Control.

[ref-9] Castañeda-Álvarez NP, Khoury CK, Achicanoy HA, Bernau V, Dempewolf H, Eastwood RJ, Guarino L, Harker RH, Jarvis A, Maxted N, Müller JV (2016). Global conservation priorities for crop wild relatives. Nature Plants.

[ref-10] Clements MA, Ellyard RK (1979). The symbiotic germination of Australian terrestrial orchids. American Orchid Society Bulletin.

[ref-11] Comby M, Lacoste S, Baillieul F, Profizi C, Dupont J (2016). Spatial and temporal variation of cultivable communities of co-occurring endophytes and pathogens in wheat. Frontiers in Microbiology.

[ref-12] Ding N, Kupper JV, McNear Jr DH (2015). Phosphate source interacts with endophyte strain to influence biomass and root system architecture in tall fescue. Agronomy Journal.

[ref-13] Druzhinina IS, Seidl-Seiboth V, Herrera-Estrella A, Horwitz BA, Kenerley CM, Monte E, Mukherjee PK, Zeilinger S, Grigoriev IV, Kubicek CP (2011). *Trichoderma*: the genomics of opportunistic success. Nature Reviews Microbiology.

[ref-14] Games PA, Howell JF (1976). Pairwise multiple comparison procedures with unequal n’s and/or variances: a Monte Carlo study. Journal of Educational Statistics.

[ref-15] Grubb PJ, Green HE, Merrifield RCJ (1969). The ecology of chalk heath: its relevance to the calcicole–calcifuge and soil acidification problems. Journal of Ecology.

[ref-16] Hajjar R, Hodgkin T (2007). The use of wild relatives for crop improvement: a survey of developments over the past 20 years. Euphytica.

[ref-17] Hale IL, Broders K, Iriarte G (2014). A Vavilovian approach to discovering crop-associated microbes with potential to enhance plant immunity. Frontiers in Plant Science.

[ref-18] Harman GE, Howell CR, Viterbo A, Chet I, Lorito M (2004). *Trichoderma* species—opportunistic, avirulent plant symbionts. Nature Reviews Microbiology.

[ref-19] Hartmann A, Schmid M, Van Tuinen D, Berg G (2009). Plant-driven selection of microbes. Plant and Soil.

[ref-20] Hughes KW, Petersen RH, Lickey EB (2009). Using heterozygosity to estimate a percentage DNA sequence similarity for environmental species’ delimitation across basidiomycete fungi. New Phytologist.

[ref-21] Jones N (2013). Food fuelled with fungi. Nature.

[ref-22] Khoury C, Laliberté B, Guarino L (2010). Trends in ex situ conservation of plant genetic resources: a review of global crop and regional conservation strategies. Genetic Resources and Crop Evolution.

[ref-23] Ko WH, Yang CH, Lin MJ, Chen CY, Tsou YJ (2011). *Humicola phialophoroides* sp nov from soil with potential for biological control of plant diseases. Botanical Studies.

[ref-24] Kokoska S, Nevison C (1989). Critical values for the studentized range distribution. Statistical tables and formulae.

[ref-25] Lakew B, Eglinton J, Henry RJ, Baum M, Grando S, Ceccarelli S (2011). The potential contribution of wild barley (*Hordeum vulgare ssp spontaneum*) germplasm to drought tolerance of cultivated barley (*H. vulgare ssp vulgare*). Field Crop Research.

[ref-26] LaMotte Chemical Products (1967). LaMotte soil handbook.

[ref-27] Lang J, Hu J, Ran W, Xu Y, Shen Q (2012). Control of cotton Verticillium wilt and fungal diversity of rhizosphere soils by bio-organic fertilizer. Biology and Fertility of Soils.

[ref-28] Larriba E, Jaime M, Nislow C, Martin-Nieto J, Lopez-Llorca LV (2015). Endophytic colonization of barley (*Hordeum vulgare*) roots by the nematophagous fungus *Pochonia chlamydosporia* reveals plant growth promotion and a general defense and stress transcriptomic response. Journal of Plant Research.

[ref-29] Lau JA, Lennon JT (2012). Rapid responses of soil microorganisms improve plant fitness in novel environments. Proceedings of the National Academy of Sciences of the United States of America.

[ref-30] López-García Å, Horn S, Rillig MC, Hempel S (2016). Spatial and niche-based ecological processes drive the distribution of endophytic Sebacinales in soil and root of grassland communities. FEMS Microbiology Ecology.

[ref-31] Malinowski DP, Belesky DP (2000). Adaptations of endophyte-infected cool-season grasses to environmental stresses: Mechanisms of drought and mineral stress tolerance. Crop Science.

[ref-32] McCouch SR, Sweeney M, Li J, Jiang H, Thomson M, Septiningsih E, Edwards J, Moncada P, Xiao J, Garris A, Tai T (2007). Through the genetic bottleneck: *O. rufipogon* as a source of trait-enhancing alleles for *O. sativa*. Euphytica.

[ref-33] Menzies JG, Ehret DL, Koch C, Bogdanoff C (1998). *Humicola fuscoatra* infects tomato roots, but is not pathogenic. European Journal of Plant Pathology.

[ref-34] Mickelbart MV, Hasegawa PM, Bailey-Serres J (2015). Genetic mechanisms of abiotic stress tolerance that translate to crop yield stability. Nature Review Genetics.

[ref-35] Mitchell RB (1989). Growing hardy orchids from seeds at Kew. Plantsman.

[ref-36] Murashige T, Skoog F (1962). A revised medium for rapid growth and bio-assays with tobacco tissue cultures. Physiologia Plantarum.

[ref-37] Murphy BR, Doohan FM, Hodkinson TR (2015). Fungal root endophytes of a wild barley species increase yield in a nutrient-stressed barley cultivar. Symbiosis.

[ref-38] Nevo E (2007). Evolution of wild wheat and barley and crop improvement: studies at the Institute of Evolution. Israel Journal of Plant Science.

[ref-39] O’Callaghan M (2016). Microbial inoculation of seed for improved crop performance: issues and opportunities. Applied Microbiology and Biotechnology.

[ref-40] Oelmuller R, Sherameti I, Tripathi S, Varma A (2009). *Piriformospora indica*, a cultivable root endophyte with multiple biotechnological applications. Symbiosis.

[ref-41] Oksanen J (2015). Vegan: an introduction to ordination. http://cran.r-project.org/web/packages/vegan/vignettes/intro-vegan.pdf.

[ref-42] Pérez-Jaramillo JE, Mendes R, Raaijmakers JM (2016). Impact of plant domestication on rhizosphere microbiome assembly and functions. Plant Molecular Biology.

[ref-43] Piper PW, Millson SH (2012). Spotlight on the microbes that produce heat shock protein 90-targeting antibiotics. Open Biology.

[ref-44] Quilliam RS, Jones DL (2012). Evidence for host-specificity of culturable fungal root endophytes from the carnivorous plant *Pinguicula vulgaris* (Common Butterwort). Mycological Progress.

[ref-45] R Core Team (2016). R: a language and environment for statistical computing.

[ref-46] Radhakrishnan R, Khan AL, Kang SM, Lee I-J (2015). A comparative study of phosphate solubilization and the host plant growth promotion ability of *Fusarium verticillioides* RK01 and *Humicola sp* KNU01 under salt stress. Annals of Microbiology.

[ref-47] Redman R, Rodriguez R (2007). The population dynamics and symbiotic lifestyle of fungal endophytes in plant hosts is driven by environmental conditions. Comparative Biochemistry and Physiology - Part A.

[ref-48] Richardson AE (2001). Prospects for using soil microorganisms to improve the acquisition of phosphorus by plants. Australian Journal of Plant Physiology.

[ref-49] Rodriguez R, Redman R (2008). More than 400 million years of evolution and some plants still can’t make it on their own: plant stress tolerance via fungal symbiosis. Journal of Experimental Botany.

[ref-50] Schauer N, Zamir D, Fernie AR (2005). Metabolic profiling of leaves and fruit of wild species tomato: a survey of the *Solanum lycopersicum* complex. Journal of Experimental Botany.

[ref-51] Tester M, Langridge P (2010). Breeding technologies to increase crop production in a changing world. Science.

[ref-52] Valentinuzzi F, Mason M, Scampicchio M, Andreotti C, Cesco S, Mimmo T (2015). Enhancement of the bioactive compound content in strawberry fruits grown under iron and phosphorus deficiency. Journal of the Science of Food and Agriculture.

[ref-53] Vassilev N, Vassileva M, Nikolaeva I (2006). Simultaneous P-solubilizing and biocontrol activity of microorganisms: potentials and future trends. Applied Microbiology and Biotechnology.

[ref-54] Wali PR, Helander M, Saloniemi I, Ahlholm J, Saikkonen K (2009). Variable effects of endophytic fungus on seedling establishment of fine fescues. Oecologia.

[ref-55] Wang Y, Thorup-Kristensen K, Stoumann Jensen L, Magid J (2016). Vigorous root growth is a better indicator of early nutrient uptake than root hair traits in spring wheat grown under low fertility. Frontiers in Plant Science.

[ref-56] White TJ, Bruns TD, Lee S, Taylor JW, Innis MA, Gelfand DH, Sninsky JJ, White TJ (1990). Amplification and direct sequencing of fungal ribosomal RNA genes for phylogenetics. PCR protocols: a guide to methods and applications.

[ref-57] Wicklow DT, Jordan AM, Gloer JB (2009). Antifungal metabolites (monorden, monocillins I, II, III) from *Colletotrichum graminicola*, a systemic vascular pathogen of maize. Mycological Research.

[ref-58] Wicklow DT, Joshi BK, Gamble WR, Gloer JB, Dowd PF (1998). Antifungal metabolites (monorden, monocillin IV, and cerebrosides) from *Humicola fuscoatra* traaen NRRL 22980, a mycoparasite of *Aspergillus flavus* sclerotia. Applied Environmental Microbiology.

[ref-59] Witcombe JR, Hollington PA, Howarth CJ, Reader S, Steele KA (2008). Breeding for abiotic stresses for sustainable agriculture. Philosophical Transactions of the Royal Society B: Biological Sciences.

[ref-60] Xiao J, Grandillo S, Ahn SN, McCouch SR, Tanksley SD (1996). Genes from wild rice improve yield. Nature.

[ref-61] Yang C-H, Lin M-J, Su H-J, Ko W-H (2014). Multiple resistance-activating substances produced by *Humicola phialophoroides* isolated from soil for control of Phytophthora blight of pepper. Botanical Studies.

[ref-62] Yao S, Guldan S (2015). Challenges of strawberry production in high-pH soil at high elevation in the southwestern United States. HortScience.

[ref-63] Yokoya K, Zettler LW, Kendon JP, Bidartondo MI, Stice AL, Skarha S, Corey LL, Knight AC, Sarasan V (2015). Preliminary findings on identification of mycorrhizal fungi from diverse orchids in the Central Highlands of Madagascar. Mycorrhiza.

[ref-64] Zakharova K, Marzban G, De Vera JP, Lorek A, Sterflinger K (2014). Protein patterns of black fungi under simulated Mars-like conditions. Scientific Reports.

[ref-65] Zhang HH, Tang M, Chen H, Wang YJ (2012). Effects of a dark-septate endophytic isolate LBF-2 on the medicinal plant *Lycium barbarum* L. Journal of Microbiology.

[ref-66] Zhou WN, White JF, Soares MA, Torres MS, Zhou ZP, Li HY (2015). Diversity of fungi associated with plants growing in geothermal ecosystems and evaluation of their capacities to enhance thermotolerance of host plants. Journal of Plant Interactions.

