# Supplementary material for: Endophytic fungal diversity of Fragaria vesca, a crop wild relative of strawberry, along environmental gradients within a small geographical area
Source: PeerJ. 2017 Jan 5;5:e2860. doi: 10.7717/peerj.2860 (PMC5289447; doi:10.7717/peerj.2860)
Supplement: Table S1 [file peerj-05-2860-s003.docx]

Supplementary Table. GenBank sequence matches and accession codes of isolated OTUs.

| OTU | From | Isolates | Closest named | | | GenBank  code |
| --- | --- | --- | --- | --- | --- | --- |
|  |  |  | Name | Identity% | Accession no. |  |
| *Dactylonectria* sp. OTU-N2 | A,B,C,D,E,F,G | 56 | *Dactylonectria alcacerensis Dactylonectria macrodidyma Dactylonectria torresensis Ilyonectria alcacerensis* | 100.0 100.0 100.0 100.0 | NR_121498 KT692589 KR019360 JF735331 | KX610377 |
|  | A,D,E,F,G | 9 | *Dactylonectria alcacerensis Dactylonectria macrodidyma Dactylonectria torresensis* | 99.8 99.8 99.8 | NR_121498 KT692589 KR019360 | KX610378 |
| *Ilyonectria* sp. OTU-N4 | A,B,E,F,G | 17 | *Ilyonectria radicicola Ilyonectria robusta* | 100.0 100.0 | KF240803 JX045819 | KX610380 |
|  | F,G | 5 | *Ilyonectria robusta Ilyonectria destructans Neonectria radicicola Cylindrocarpon* sp. | 100.0 100.0 100.0 100.0 | KR019364 KF915980 GU479904 AJ279490 | KX610381 |
| *Ilyonectria* sp. OTU-N5 | A | 1 | *Ilyonectria radicicola Ilyonectria rufa* | 99.4 99.4 | KC989076 JF735278 | KX610382 |
|  | A,B,E,G | 19 | *Ilyonectria radicicola Ilyonectria rufa* | 100.0 100.0 | KC989076 JF735278 | KX610383 |
|  | A,B,F,G | 11 | *Ilyonectria crassa Neonectria radicicola Neonectria radicicola* | 100.0 100.0 100.0 | KJ475469 HQ840390 GU934548 | KX610384 |
| *Fusarium* sp. OTU-F1 | C,D | 5 | *Fusarium tricinctum Fusarium avenaceum* | 100.0 100.0 | KT692586 KT004586 | KX610388 |
|  | G | 7 | *Fusarium avenaceum Fusarium tricinctum* | 100.0 100.0 | KP292814 GQ922562 | KX610389 |
| *Phomopsis columnaris* | A,C,D,E,F | 44 | *Phomopsis columnaris* | 100.0 | JX045820 | KX610404 |
|  | A,C,D,E,F | 22 | *Phomopsis columnaris* | 100.0 | KC145883 | KX610405 |
| *Phomopsis* sp. | B,C,E | 39 | *Phomopsis* sp. | 100.0 | KT264444 | KX610406 |
| *Monodictys* sp. | E | 3 | *Monodictys* sp. | 99.3 | KC427051 | KX610407 |
|  | D,E | 2 | *Monodictys* sp. | 98.0 | KC427051 | KX610408 |
|  | B | 1 | *Monodictys* sp. | 98.5 | KC427051 | KX610409 |
| *Cadophora* sp. A | A | 1 | *Cadophora* sp. | 99.8 | KT270219 | KX610415 |
|  | G | 1 | *Cadophora* sp. | 99.3 | KT269835 | KX610416 |
|  | C,G | 2 | *Cadophora* sp. | 99.7 | KT269095 | KX610417 |
|  | G | 1 | *Cadophora* sp. | 99.8 | KT269095 | KX610418 |
|  | F | 1 | *Cadophora* sp. | 100.0 | KT269095 | KX610419 |
| *Cadophora* sp. B | B | 1 | *Cadophora* sp. | 100.0 | JN859246 | KX610420 |
|  | B,E | 5 | *Cadophora* sp. | 100.0 | JN859252 | KX610421 |
|  | B,E | 2 | *Cadophora* sp. | 100.0 | JN859258 | KX610422 |
|  | B | 1 | *Cadophora* sp. | 99.8 | JN859252 | KX610423 |
|  | B | 1 | *Cadophora* sp. | 99.8 | JN859240 | KX610424 |
|  | F | 1 | *Cadophora* sp. | 99.8 | JN859236 | KX610425 |
|  | F | 1 | *Cadophora* sp. | 100.0 | FJ666349 | KX610426 |
| Helotiaceae A | B,D,G | 7 | Helotiales sp. | 100.0 | KT269568 | KX610432 |
|  | B,C,F,G | 8 | Helotiales sp. | 100.0 | KT269664 | KX610433 |
|  | B | 1 | Helotiales sp. | 99.8 | KT269664 | - |
|  | F | 1 | Helotiales sp. | 99.4 | KT269664 | KX610434 |
| *Ceratobasidium albasitensis* OTUrA | B,D,G | 31 | *Ceratobasidium albasitensis* | 99.8 | HQ680963 | KX610453 |
| *Ceratobasidium* OTUrD | A | 6 | *Ceratobasidium* sp. | 99.6 | FN812725 | KX610456 |
|  | B | 5 | *Ceratobasidium* sp. | 99.3 | AB290022 | KX610457 |
|  | C | 1 | *Ceratobasidium* sp. | 99.6 | AB290022 | KX610458 |
| *Rhizoctonia butinii* OTUrB | A | 4 | *Rhizoctonia butinii* | 94.7 | KP334098 | KX610454 |
| *Cryptosporiopsis* sp. | A,B | 5 | *Pezicula melanigena* | 99.8 | KR859211 | KX610430 |
| *Fusarium oxysporum* OTU-F3 | C | 3 | *Fusarium oxysporum Fusarium solani* | 100.0 100.0 | LN828196 KR708647 | KX610391 |
|  | C | 1 | *Fusarium oxysporum* | 100.0 | KT921204 | KX610392 |
|  | A | 2 | *Fusarium oxysporum* | 100.0 | LN828172 | KX610393 |
| *Dactylonectria macrodidyma* OTU-N1 | A,D | 3 | *Dactylonectria macrodidyma* | 100.0 | HM036602 | KX610376 |
| *Bionectria ochroleuca* OTU-B1 | A | 1 | *Clonostachys rosea Bionectria ochroleuca* | 100.0 100.0 | KT921200 JQ782651 | KX610396 |
| *Trichosporon porosum* | A | 1 | *Trichosporon porosum* | 100.0 | NR_073209 | KX610459 |
| *Neonectria* sp. OTU-N3 | B | 1 | *Neonectria* sp. | 100.0 | KF428615 | KX610379 |
| *Truncatella angustata* | B | 2 | *Truncatella angustata* | 100.0 | KT582088 | KX610411 |
| Pleosporales | B | 1 | Pleosporales sp. | 94.3 | JN859337 | KX610436 |
| *Mortierella verticillata* | B | 1 | *Mortierella verticillata* | 100.0 | KF944471 | KX610449 |
| *Cryptosporiopsis radicicola* | C,F | 10 | *Pezicula radicicola Cryptosporiopsis radicicola* | 100.0 100.0 | KR859236 KC311507 | KX610431 |
| *Cylindrocarpon olidum* OTU-N7 | C,D | 2 | *Cylindrocarpon* sp. | 100.0 | KT269448 | KX610386 |
|  | D | 1 | *Cylindrocarpon* sp. | 99.8 | KT269448 | KX610387 |
| *Periconia macrospinosa* | C | 1 | *Periconia macrospinosa* | 100.0 | JN859365 | KX610410 |
| *Glarea* sp. | C | 1 | *Glarea* sp. | 99.8 | KT268651 | KX610435 |
| *Pyrenochaeta* sp. | C | 1 | *Pyrenochaeta* sp. | 100.0 | KT268458 | KX610437 |
| *Fusarium* sp.OTU-F5 | D,G | 2 | *Fusarium* sp. | 100.0 | AY633561 | KX610395 |
| *Trichothecium croticinigenum* | D | 2 | *Acremonium crotocinigenum* | 99.3 | GU934498 | KX610402 |
| *Cylindrodendrum alicantinum* OTU-N6 | D | 2 | *Cylindrodendrum alicantinum* | 100.0 | KP456014 | KX610385 |
| *Paraphoma* sp. | E,G | 2 | *Paraphoma* sp. | 100.0 | KT269147 | KX610439 |
| *Gliomastix murorum* OTU-G1 | E | 1 | *Gliomastix murorum* | 100.0 | HQ115690 | KX610397 |
| *Chaetomium nigricolor* | E | 1 | *Chaetomium nigricolor* | 100.0 | EU543258 | KX610413 |
| *Pyrenochaeta* sp. | E | 2 | *Pyrenochaeta* sp. | 100.0 | KF636767 | KX610438 |
| *Mortierella gamsii* | E | 1 | *Mortierella* sp. | 99.8 | JX270447 | KX610447 |
| *Mucor saturninus* | E | 1 | *Mucor saturninus* | 99.4 | NR_103635 | KX610450 |
| *Mucor fragilis* | E | 1 | *Mucor fragilis* | 99.3 | KU319073 | KX610451 |
| *Trichoderma harzianum* OTU-T1 | F | 7 | *Trichoderma harzianum* | 100.0 | JF311961 | KX610398 |
| *Trichoderma alni* OTU-T2 | F | 1 | *Trichoderma alni* | 100.0 | EU518654 | KX610399 |
| *Trichoderma koningiopsis* OTU-T3 | F | 2 | *Trichoderma koningiopsis Trichoderma petersenii* | 100.0 100.0 | KR995115 KJ783288 | KX610400 |
| *Daldinia concentrica* | F | 1 | *Daldinia concentrica* | 100.0 | KC692211 | KX610412 |
| *Humicola* sp. | F | 1 | *Humicola* sp. | 99.2 | HQ637372 | KX610414 |
| *Cadophora luteo-olivacea* | F | 2 | *Cadophora luteo-olivacea* | 100.0 | HM116747 | KX610427 |
| Helotiales A | F | 1 | *Phialea strobilina* | 93.9 | EF596821 | KX610428 |
| *Herpotrichia* | F | 1 | *Herpotrichia* sp. | 99.6 | KT270238 | KX610440 |
| *Knufia* sp. | F | 1 | *Knufia tsunedae* | 99.4 | NR_132842 | KX610444 |
| *Ceratobasidium* OTUrC | F | 2 | *Rhizoctonia* sp. | 99.8 | AY842391 | KX610455 |
| *Volutella rosea* OTU-V1 | G | 5 | *Volutella rosea* | 100.0 | KM231769 | KX610461 |
| Helotiales B | G | 1 | Helotiales sp. | 100.0 | JN859270 | KX610429 |
| *Stachybotrys* sp. | G | 1 | *Stachybotrys longispora Stachybotrys chartarum* | 90.4 90.5 | AF081482 KC305339 | KX610401 |
| *Fusarium* sp. OTU-F2 | G | 2 | *Fusarium acuminatum Fusarium tricinctum Fusarium avenaceum* | 100.0 100.0 100.0 | KT192260 KT192246 KP265365 | KX610390 |
| *Fusarium redolens* OTU-F4 | G | 1 | *Fusarium redolens Fusarium oxysporum Fusarium acuminatum* | 100.0 100.0 100.0 | KP942907 KJ001182 JX114782 | KX610394 |
| *Plectosphaerella cucumerina* | G | 1 | *Plectosphaerella cucumerina* | 98.7 | KP942934 | KX610403 |
| *Didymella vitalbina* | G | 1 | *Didymella* sp. *Didymella vitalbina* | 100.0 100.0 | KT309898 FJ515592 | KX610441 |
| *Phoma* sp. | G | 1 | *Phoma multirostrata Boeremia exigua Boeremia foveata* | 100.0 100.0 100.0 | KF428213 KT193802 JQ804843 | KX610442 |
| *Alternaria* sp. | G | 1 | *Alternaria triticina* | 100.0 | JX418360 | KX610443 |
| *Exophiala* sp. | G | 1 | *Exophiala salmonis* | 95.8 | AY213652 | KX610445 |
| *Tetracladium maxiliforme* | G | 1 | *Tetracladium maxiliforme* | 100.0 | DQ068996 | KX610446 |
| *Mortierella zonata* | G | 1 | *Mortierella zonata* | 100.0 | KF944462 | KX610448 |
| *Absidia glauca* | G | 3 | *Absidia glauca* | 99.8 | JN205820 | KX610452 |
| *Pythium* sp. | G | 1 | *Pythium* sp *Pythium attrantheridium* | 99.5 99.5 | EU038829 KJ744316 | KX610460 |
